# Risk factors for mortality among kidney transplant recipients with COVID-19 in Saudi Arabia: a case-control study

**DOI:** 10.1186/s12879-025-10994-4

**Published:** 2025-04-18

**Authors:** Dema A. Alissa, Rawan Almasuood, Baraa Alghalyini, Hala Tamim, Mohammed Alaa Raslan, Abdul Rehman Zia Zaidi, Bader Abou Shaar, Mhd Malek Al Masri, Adnan Abdul Karim, Wejdan Aburas, Waled Aburas, Ahmed H. Al-jedai, Reem S. Almaghrabi

**Affiliations:** 1 Therapeutic Affairs Deputyship, Ministry of Health, Riyadh, Saudi Arabia; 2 Colleges of Medicine and Pharmacy, Al-Faisal University, Riyadh, Saudi Arabia; 3 College of Pharmacy, Princess Nourah bint Abdulrahman University, Riyadh, Saudi Arabia; 4 Organ Transplant Centre of Excellence, King Faisal Specialist Hospital and Research Center, Riyadh, Saudi Arabia; 5 Department of Family and Community Medicine, College of Medicine, Alfaisal University, Riyadh, Saudi Arabia; 6 Department of Kinesiology and Health Science, York University, Toronto, ON Canada; 7 College of Pharmacy, University of Hail, Hail, Saudi Arabia

**Keywords:** COVID-19, Mortality, Kidney transplant recipients, Saudi Arabia, Risk factors

## Abstract

**Background:**

The COVID-19 pandemic, caused by the novel coronavirus SARS-CoV-2, has profoundly impacted global health, leading to over 74 million confirmed cases and 1.67 million deaths by December 2020. In Saudi Arabia, extensive measures were implemented to mitigate the spread of the virus. Kidney transplant recipients, due to their immunosuppressed status, are particularly vulnerable to severe COVID-19 outcomes. This study aims to identify risk factors associated with mortality in COVID-19-infected kidney transplant patients in Saudi Arabia. The primary objective is to identify mortality risk factors among COVID-19-infected kidney transplant patients. The secondary objective is to compare clinical management and outcomes between deceased and recovered patients.

**Methodology:**

This case-control study matched 82 deceased kidney transplant patients (cases) with 151 survivors (controls). Data were collected from the National Registry for COVID-19 Mortality and King Faisal Specialist Hospital and Research Centre (KFSH&RC) for patients diagnosed between March 2020 and January 2021. Key variables included demographic information, comorbidities, clinical symptoms, and treatment details. Statistical analyses involved chi-square tests and multivariable logistic regression to assess associations with mortality.

**Results:**

Among cases, 93.9% required ICU admission, and 95.1% were intubated. Males constituted 73.2% of cases, with 53.7% aged over 60. Cardiovascular comorbidities were more prevalent among cases (97% vs. 87.4%, *p* = 0.01). and presented more frequently with fever, cough, and respiratory distress. In multivariable analysis, fever, shortness of breath, and desaturation were associated with increased mortality odds. Notably, patients who discontinued immunosuppressive therapy had higher mortality odds (OR = 63.2, *p* = 0.083), whereas those who held or adjusted their therapy had significantly lower odds (OR = 0.1, *p* = 0.042; OR = 0.0, *p* = 0.007). Bacterial infections also increased mortality risk (OR = 56.6, *p* = 0.009).

**Conclusion:**

This study identifies critical risk factors for mortality among kidney transplant patients infected with COVID-19 in Saudi Arabia. The findings underscore the need for tailored clinical management strategies to improve outcomes in this vulnerable population. Further research is warranted to explore long-term implications and effective treatment protocols.

## Introduction/Background

In December 2019, a novel coronavirus was identified as the causative agent of a cluster of pneumonia cases in Wuhan (Hubei, China), namely severe acute respiratory syndrome coronavirus 2 (SARS-CoV-2). In February 2020, the World Health Organization (WHO) named the disease associated with SARS-CoV-2 infections as coronavirus “COVID-19”. COVID-19 rapidly evolved into a global pandemic, with over 74 million confirmed cases and 1.67 million deaths reported to WHO by December 2020.The outline of disease prognosis [1]. In the face of this challenge, the Kingdom of Saudi Arabia (KSA) curbed the COVID-19 spread by multiple approaches such as limiting international traveling, quarantining, studying from home, setting rules for social distancing, and wearing masks in public places, free screening, and treatment of COVID-19 as well as increasing the hospitals’ capacity and increasing the public awareness toward this pandemic [2].

The disease prognosis varies, with approximately 80% of cases resolving, while others require hospitalization and supplemental oxygen within 7–10 days of symptom onset [3]. Nonetheless, risk factors such as age, obesity, hypertension, diabetes, cancer patients, chronic kidney disease, and immunocompromised are all linked to progression and mortality. Solid organ transplant (SOT) recipients are particularly susceptible to severe outcomes from COVID-19 due to their immunosuppressed state, a consequence of the necessary immunosuppressive therapies used to prevent organ rejection. This immunosuppression, combined with frequent healthcare interactions, increases their risk of severe complications and mortality compared to the general population [4, 5].

Research has shown that SOT patients with COVID-19 are at a significantly higher risk of severe disease and mortality. A Spanish cohort study reported a 27% mortality rate among SOT patients, with lung transplantation, age over 60, and hospital-acquired infections identified as significant risk factors [6]. Additionally, studies on kidney transplant recipients (KTRs) have highlighted high rates of severe symptoms such as fever and respiratory distress, with mortality rates reaching 26.3% [7]. Comparative analyses have indicated that while overall mortality rates between SOT and non-SOT patients might not differ significantly, SOT patients are more likely to experience severe disease due to their immunosuppressive regimens and underlying health conditions [8, 9].

Managing immunosuppressive therapy during COVID-19 presents unique challenges. Adjustments in these regimens, including reduction or discontinuation, have been common to mitigate severe outcomes [4]. Recent studies have shown that early intervention with antiviral therapies and monoclonal antibodies significantly reduces the risk of severe disease progression in SOT patients, emphasizing the importance of timely and targeted treatment strategies [5, 10].

Despite the increasing volume of research, there remains a significant gap in comprehensive studies focusing on the specific risk factors contributing to mortality among COVID-19-infected kidney transplant patients, particularly in the Middle East. This study aims to address this gap by evaluating the risk factors associated with mortality in COVID-19-infected kidney transplant patients in Saudi Arabia, utilizing data from the national registry and King Faisal Specialist Hospital and Research Centre (KFSH&RC).

### Objectives

#### Primary objective

To identify the risk factors associated with mortality among COVID-19-infected kidney transplant patients in Saudi Arabia.

#### Secondary objective

To compare the clinical management and outcomes of deceased and recovered kidney transplant patients with COVID-19.

## Methodology

### Study design

This study employed a case-control design to identify risk factors associated with mortality among COVID-19-infected kidney transplant patients in Saudi Arabia matched in a 1:2 ratio with recovered SOT patients. Cases refer to deceased kidney transplant patients with COVID-19, while controls represent survivors.

### Study population

Data was collected from the National Registry for COVID-19 Mortality and the KFSH&RC. Inclusion criteria include:

- Kidney transplant recipients.
- Diagnosed with COVID-19 between March 2020 until January 2021.
- Recorded in the National Registry for COVID-19 Mortality and/or treated at KFSH&RC.

### Data collection

Patient data was extracted from electronic health records and the national registry. The data collected includes:

- Demographic information (age, gender, nationality, BMI).
- Clinical data (comorbidities, symptoms, laboratory findings).
- Transplant details (type of organ, date of transplant, immunosuppressive regimen).
- COVID-19 diagnosis and course (symptoms, severity, treatments, ICU admission, intubation).
- Outcomes (recovery, mortality).

### Variables

The primary outcome is mortality due to COVID-19. Key variables to be analyzed include:

- Demographic factors: age, gender, Body Mass Index (BMI), nationality.
- Clinical factors: presence of comorbidities (e.g., diabetes, hypertension, cardiovascular diseases), symptom severity, laboratory markers (e.g., lymphocyte count, serum potassium levels).
- Treatment factors: type of immunosuppressive therapy, antiviral treatments, monoclonal antibody treatments, ICU admission, mechanical ventilation.

### Statistical analysis

Descriptive statistics were used to summarize the characteristics of cases and controls. Chi-square statistics were performed to identify the association between the different socio-demographic, symptom, and infection-related factors with mortality. A multivariable logistic regression model was performed to identify independent factors associated with mortality after adjusting for confounders. Adjusted Odds Ratios (OR) and 95% confidence intervals (CI) were reported.

## Results

The study comprised 82 cases (kidney transplant patients who died from COVID) and 151 controls (kidney transplant patients who did not die from COVID). ICU admission was significantly higher among cases (93.9%) compared to controls (19.5%, *p* < 0.001), with intubation rates also markedly greater (95.1% vs. 2.4%, *p* < 0.001), and 95.1% were intubated, compared to 2.4% among the controls (*p* < 0.001). Table 1 shows the socio-demographic, symptom-related, and infection-related characteristics of cases and controls. Males made up 73.2% of the cases compared to 55% of the controls (*p* = 0.006), and 53.7% of the cases were over 60 years of age compared to 28.7% among controls, *p* < 0.001. Cases had a greater proportion of cardiovascular comorbidities than controls (97% versus 87.4%, *p* = 0.01). Furthermore, the proportion of those with cough, fever, shortness of breath, and desaturation were significantly higher among cases than controls, but diarrhea was significantly lower among cases.

**Table 1:** Relationship between death and socio-demographic, symptom-related, and infection-related factors among study participants

	Total	Cases	Controls	
	**N (%)**	**N (%)**	**N (%)**	**P-value**
**Socio-demographic**				
**Sex**				
Male	143 (61.4)	60 (73.2)	83 (55.0)	**0.006**
**Nationality**				
Saudi	218 (93.6)	67 (81.7)	151 (100.0)	**< 0.001**
**Age**				
*≤* 20	8 (3.4)	0 (0)	8 (5.3)	**< 0.001**
20.1–60	137 (58.8)	38 (46.3)	99 (66.0)	
> 60	87 (37.3)	44 (53.7)	43 (28.7)	
**Comorbidities**				
Cardiovascular	212 (91.0)	80 (97.6)	132 (87.4)	**0.01**
Endocrine	189 (81.1)	72 (87.8)	117 (77.5)	0.055
Renal	184 (79.0)	63 (76.8)	121 (80.1)	0.555
Neurologic	143 (61.4)	49 (59.8)	94 (62.3)	0.709
Hepatic	142 (60.9)	51 (62.2)	91 (60.3)	0.773
Thromboembolic	139 (59.7)	51 (62.2)	88 (58.3)	0.561
Respiratory	139 (59.7)	50 (61.0)	89 (58.9)	0.762
**Symptom Development**				
Cough	118 (50.6)	55 (67.1)	63 (41.7)	**< 0.001**
Fever	110 (47.2)	54 (65.9)	56 (37.1)	**< 0.001**
Shortness of breath	105 (45.1)	70 (85.4)	35 (23.2)	**< 0.001**
Desaturation	61 (26.2)	57 (69.5)	4 (2.6)	**< 0.001**
Diarrhea	38 (16.3)	8 (9.8)	30 (19.9)	**0.046**
**Change in Immunosuppressant Treatment**				
No	30 (12.9)	12 (14.8)	18 (12.6)	**< 0.001**
Only holding	86 (36.9)	24 (29.6)	62 (43.4)	
Only discontinuing	19 (8.2)	18 (22.2)	1 (0.7)	
Combination of changes	69 (29.6)	11 (13.6)	58 (40.6)	
Other*	20 (8.6)	16 (19.8)	4 (2.8)	
**Co-infection**				
Bacterial	32 (13.7)	25 (30.5)	7 (4.6)	**< 0.001**

*Patients with changes limited to only switching (*n* = 6), only decreasing (*n* = 9%) and only increasing (*n* = 5)

Table 2 shows the findings of the multivariable logistic regression model. None of the sociodemographic or comorbidities were significantly associated with mortality; however, those who had fever, shortness of breath, or desaturation were significantly at increased odds of dying, and those who reported having diarrhea were significantly at reduced odds of dying. In terms of Immunosuppressant Treatment Change, when compared to those with “no change”, those who “only discontinued” were at a higher odds of dying, with results approaching significance (OR = 63.2, 95% CI=(0.6-6814.5), *p* = 0.083), whereas those who “only held” and those who made a “combination of changes” were significantly at a lower odds of dying (OR = 0.1, 95% CI (0.0-0.9), *p* = 0.042 and OR = 0.0, 95% CI (0.0-0.3), *p* = 0.007 respectively). In addition, patients with bacterial infection were at increased odds of dying when compared to those without (56.6 (2.8-1160.8), *p* = 0.009).

**Table 2:** Results of the multivariable logistic regression

	Adjusted OR (95% CI)	*P*-value
**Sex**		
Male	1.8 (0.3–10.2)	0.527
Female	1	
**Age**		
*≤* 20	-	
20.1–60	0.9 (0.2–4.8)	0.879
> 60	1	
**Comorbidities**		
Cardiovascular	10.1 (0.2-557.6)	0.259
Endocrine	3.3 (0.1–76.0)	0.459
Renal	1.2 (0.1–14.9)	0.903
Neurologic	15.1 (0.3-776.4)	0.177
Hepatic	1.2 (0.0-42.5)	0.908
Thromboembolic	0.1 (0.0-5.2)	0.253
Respiratory	0.3 (0.0-266.7)	0.749
**Symptom Development**		
Cough	0.3 (0.0-1.8)	0.176
Fever	46.3 (4.0-530.1)	**0.002**
Shortness of breath	56.9 (6.5–495.0)	**< 0.001**
Desaturation	547.8 (39.8-7537.1)	**< 0.001**
Diarrhea	0.0 (0.0-0.4)	**0.008**
**Change in Immunosuppressant Treatment**		
No	1	
Only holding	0.1 (0.0-0.9)	**0.042**
Only discontinuing	63.2 (0.6-6814.5)	0.083
Combination of changes	0.0 (0.0-0.3)	**0.007**
Other*	2.6 (0.1–61.2)	0.543
**Co-infection**		
Bacterial	56.6 (2.8-1160.8)	**0.009**

*Patients with changes limited to only switching (*n* = 6), only decreasing (*n* = 9%) and only increasing (*n* = 5)

## Discussion

Our study investigated the risk factors for mortality in kidney transplant patients diagnosed with COVID-19. Our findings indicate that fever, shortness of breath, and hypoxia significantly increased mortality risk. Additionally, discontinuing immunosuppressant therapy and bacterial co-infection were associated with higher mortality rates. Diarrhea was associated with lower mortality risk, highlighting an intriguing finding. This association may indicate that gastrointestinal symptoms reflect a more robust immune response or altered viral dynamics, potentially resulting in improved patient outcomes. Further research is warranted to elucidate the mechanisms underlying this relationship in the context of COVID-19 severity [11]. There were no studies from Saudi Arabia that investigated the risk factors for mortality among the solid organ transplant population. However, some studies looked into the risk factors of mortality in the general population. The risk factors for in-hospital mortality among COVID-19 patients in Saudi Arabia, included older age, the presence of chronic kidney disease, acute respiratory distress syndrome (ARDS), diabetes mellitus, hypertension, heart failure, ischemic heart disease, and acute kidney injury [12–14]. Solid Organ Transplant recipients are at an increased mortality risk across the pandemic. A study from the Japan Transplant Society investigated the rate of mortality of SOT recipients with COVID-19 in relation to the dominant variant of concern (VOC) by calculating the standardized mortality rate (SMR) [15]. The study showed that SOT recipients had excess SMR across the pandemic with all VOC but highest with Omicron subvariants (BA.1/BA.2) and Omicron BA.5 [15]. This finding underscores the need for continued investigation into the risk factors for severe disease and mortality in this vulnerable population. It also highlights the importance of ongoing protective measures, such as vaccine boosters and immunosuppression optimization, particularly for older SOTRs, to mitigate the risks associated with COVID-19 infection [15]. Several studies have looked into the risk factors for mortality in kidney transplant patients, high oxygen requirement and need for mechanical ventilation, co-infection, high inflammatory markers, and anemia were found to be associated with a higher risk for mortality [16–20]. Similar findings were reported in a systematic review that investigated the outcome of kidney transplant patients with COVID-19 [20]. Advanced age, medical comorbidities, deceased organ transplant, dyspnea, intubation, gastrointestinal symptoms, and elevated inflammatory markers were shown to be associated with a higher risk of mortality [21].

Details on the patient’s laboratory investigations could not be obtained for our study of the cases and therefore we did not look into the relation of inflammatory markers or changes in the white blood counts to the outcome which is one of the limitations of this study.

Our results showed the importance of careful management of the immunosuppressant medication. The changes in immunosuppressant medication can significantly affect the outcomes of kidney transplant patients, particularly in the context of COVID-19. A study from Colombian transplantation centers found that kidney transplant recipients with COVID-19 who were on calcineurin inhibitors (CNI) had a higher risk of mortality compared to those on CNI-free regimens. This may suggest that the type of immunosuppression may influence outcomes [22]. Some recommendations for the management of immunosuppressant medications included reducing or discontinuing certain immunosuppressants, such as mycophenolate or mTOR inhibitors while maintaining calcineurin inhibitors and corticosteroids at minimal effective doses.Others recommended a more specific approach for the management of immunosuppressants in the setting of COVID-19 in kidney transplant patients that involved a 50% reduction in antimetabolite dose or complete withdrawal on a case-by-case basis. Stop mTOR inhibitors and maintain CNI and steroid doses for non-hospitalized patients or those with mild symptoms. While for hospitalized patients stopping antimetabolites, mTOR inhibitors, and belatacept, and maintain steroids and CNI at lower levels [23]. Our results showed that discontinuation of immunosuppressant medication was associated with higher odds for mortality compared with those who had a combination of changes in the immunosuppressant medications.

This suggests that the best approach to managing immunosuppression in kidney transplant patients with COVID-19 involves a careful balance between preventing graft rejection and minimizing the risk of over-immunosuppression, which could exacerbate the viral infection.

Finally, bacterial co-infection was associated with an increased risk of mortality in our study. Similar findings were reported by Shafiekhani et al. [24]. The study included 66 SOT patients with confirmed SARS-CoV-2 infection by RT-PCR. The findings revealed that 21.2% of the patients experienced at least one episode of co-infection, which was associated with significantly higher mortality rates [24]. Immunosuppression status may have contributed to the risk of co-infection, but a systematic review showed that around 23% of hospitalized COVID-19 patients had bacterial or fungal co-infection [25]. Proper diagnosis and management of co-infection is necessary to avoid antimicrobial overuse.

The study’s limitations include its retrospective design, which may introduce data collection and interpretation biases. The reliance on electronic health records and national registry data can result in incomplete or inconsistent information regarding comorbidities and treatment regimens. Additionally, the Saudi Arabian setting should be acknowledged, as it may limit the generalizability of the findings to other populations.

The relatively small sample size poses a significant limitation, potentially impacting the statistical power and precision of the estimates. Replication of the study with a larger cohort would strengthen the conclusions drawn. Furthermore, we recommend conducting multi-center studies to validate these findings across diverse populations.

Moreover, the missing laboratory data need to be addressed and discussed, as this limitation may have influenced the analysis and interpretation of results. Exploring additional potential confounding factors not included in the current analysis, such as specific comorbidities and details of the COVID-19 illness course, is essential. Finally, extending the analysis to include long-term outcomes and complications of COVID-19 in kidney transplant recipients would provide a more comprehensive understanding of the disease’s impact on this vulnerable population.

## Conclusion

This study highlights critical risk factors associated with mortality among COVID-19-infected kidney transplant recipients in Saudi Arabia. The findings indicate that symptoms such as fever and shortness of breath significantly increase mortality risk, while the management of immunosuppressive therapy plays a crucial role in patient outcomes. The high prevalence of cardiovascular comorbidities among deceased patients further highlights the need for vigilant monitoring and tailored interventions for this vulnerable population. These findings highlight the critical need for early identification and tailored management of high-risk patients, alongside ongoing research to optimize treatment strategies and improve survival in kidney transplant recipients with COVID-19.

## Data Availability

Availability of data and material: All data and materials are available upon request. Organs were not procured from prisoners and full reports about the institution that the organs were procured are available and includes full information on both donors and recipients.

## Abbreviations

ARDS: Acute Respiratory Distress Syndrome
BMI: Body Mass Index
CI: Confidence Intervals
KFSH&RC: King Faisal Specialist Hospital and Research Centre
KSA: Kingdom of Saudi Arabia
KTRs: Kidney Transplant Recipients
OR: Odds Ratios
SARS-CoV-2: Severe Acute Respiratory Syndrome Coronavirus 2
SMR: Standardized Mortality Rate
SOT: Solid organ transplant
VOC: Variant of Concern
WHO: World Health Organization
